# RNA interference screening identifies a novel role for PCTK1/CDK16 in medulloblastoma with c-Myc amplification

**DOI:** 10.18632/oncotarget.2699

**Published:** 2014-11-06

**Authors:** Paulina Ćwiek, Zaira Leni, Fabiana Salm, Valeriya Dimitrova, Beata Styp-Rekowska, Gianpaolo Chiriano, Michael Carroll, Katrin Höland, Valentin Djonov, Leonardo Scapozza, Patrick Guiry, Alexandre Arcaro

**Affiliations:** ^1^ Division of Pediatric Hematology/Oncology, Bern University Hospital, Bern, Switzerland; ^2^ Institute of Anatomy, University of Bern, Bern, Switzerland; ^3^ Pharmaceutical Biochemistry, School of Pharmaceutical Sciences, University of Geneva, University of Lausanne, Geneva, Switzerland; ^4^ Centre for Synthesis and Chemical Biology, University College Dublin, Belfield, Dublin, Ireland

**Keywords:** c-Myc, medulloblastoma, PCTK1, RNA interference, synthetic lethality

## Abstract

Medulloblastoma (MB) is the most common malignant brain tumor in children and is associated with a poor outcome. *cMYC* amplification characterizes a subgroup of MB with very poor prognosis. However, there exist so far no targeted therapies for the subgroup of MB with *cMYC* amplification. Here we used kinome-wide RNA interference screening to identify novel kinases that may be targeted to inhibit the proliferation of c-Myc-overexpressing MB. The RNAi screen identified a set of 5 genes that could be targeted to selectively impair the proliferation of c-Myc-overexpressing MB cell lines: *AKAP12 (*A-kinase anchor protein), *CSNK1α1* (casein kinase 1, alpha 1), *EPHA7* (EPH receptor A7) and *PCTK1* (PCTAIRE protein kinase 1). When using RNAi and a pharmacological inhibitor selective for PCTK1, we could show that this kinase plays a crucial role in the proliferation of MB cell lines and the activation of the mammalian target of rapamycin (mTOR) pathway. In addition, pharmacological PCTK1 inhibition reduced the expression levels of c-Myc. Finally, targeting PCTK1 selectively impaired the tumor growth of c-Myc-overexpressing MB cells *in vivo*. Together our data uncover a novel and crucial role for PCTK1 in the proliferation and survival of MB characterized by *cMYC* amplification.

## INTRODUCTION

Medulloblastoma (MB), a tumor arising in the cerebellum or medulla is the most prevalent malignant brain tumor in children [1]. Current treatments including surgery, craniospinal radiotherapy and chemotherapy have improved survival rates, which nowadays are approximately 80% [2, 3]. Nevertheless, there are still numerous patients with poor prognosis and survivors suffer from diminished quality of life caused by the aggressive therapy [2]. The current classification system for medulloblastoma is based on morphology (histopathology), and include variants such as desmoplastic/nodular, MBEN (medulloblastoma with extensive nodularity), classic medulloblastoma, large cell, and anaplastic medulloblastoma [4, 5]. During the past few years, transcriptome-based, molecular studies on cohorts of MB have depicted this tumor not as a single disease, but as a cluster of individual molecular subgroups. Four subtypes have been proposed with distinct characteristics in terms of gene expression, mutational profiles, epidemiology, and prognosis: Wnt, Shh, Group 3, and Group 4 [3, 6-8]. The most aggressive MB subtype (Group 3), that consists of ‘classical’ medulloblastomas and partially of the large cells/anaplastic (LCA) tumors is associated with amplification in *cMyc* [4]. Current treatments fail to cure two thirds of patients in this particular group [8]. Thus, it remains of great interest to investigate which role c-Myc plays in the biology of MB.

c-Myc, a potent and frequently deregulated oncogene in human cancers is known to promote tumor formation and metastasis by regulating cell proliferation, differentiation, angiogenesis and metabolism. Elevated c-Myc expression in tumors can occur through various mechanisms such as: amplification, chromosomal translocation, single nucleotide polymorphism (SNP) or mutation in c-Myc-related pathways [9]. Previous reports have shown that c-Myc overexpression in cancers, is highly correlated with those genes and pathways, which might not be essential in the context of normal cells [10]. Additionally, as a transcription factor without evident druggable domain and regulating essential processes in proliferative tissues, c-Myc itself may not be an appropriate drug target [11]. A large body of evidence has revealed synthetic lethality as a possible therapeutic approach to target cancer cells harbouring specific oncogenic drivers, while sparing normal cells [12, 13]. This alternative approach appears to be a useful tool to identify target molecules that in combination with c-Myc oncogenic pathways result in synthetic lethality in medulloblastoma.

## RESULTS

### High throughput siRNA screen (HTS) discovering synthetic lethality in MB

**Figure 1:**
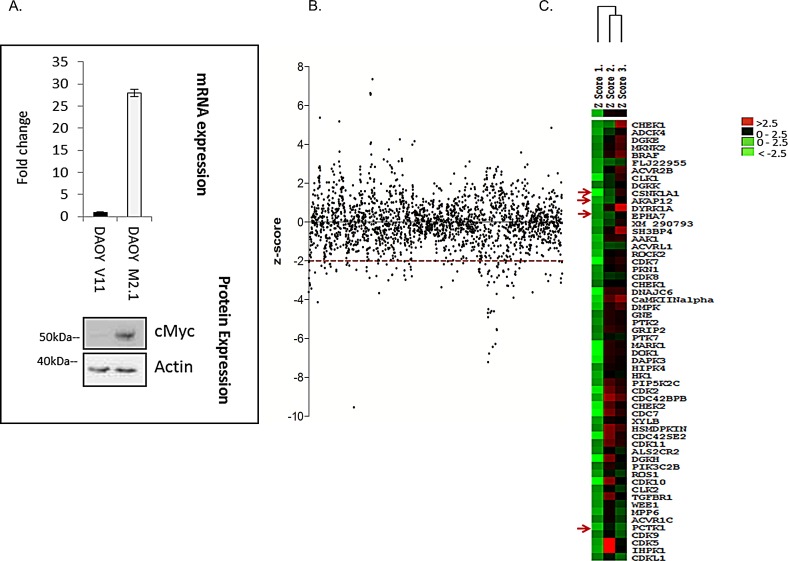
High-throughput lipid and protein kinase siRNA screen aimed at identifying drug targets for cMyc-overexpressing medulloblastoma cells A. Cell line model used for the screen: DAOY V11 transfected with empty vector and DAOY M2.1 over-expressing c-Myc (mRNA and protein level). B. Scatter plot of z-scores from the screen. C. Heat map of the top candidates from the screen.

To broadly identify genes that are essential for the survival of c-Myc-overexpressing MB cells, we performed a high-throughput siRNA screen targeting protein and lipid kinases in two clones of medulloblastoma DAOY cells: empty-vector-transfected (V11) or transfected with a c-Myc overexpressing construct (M2.1) [14] (Fig. 1A). Both clones were transfected with a kinome-wide library containing 3 distinct siRNAs targeting the same gene. Statistical analysis indicated which genes when silenced led to decreased viability of c-Myc-overexpressing cells in comparison to those without overexpression. The outcome of the screen is represented as a z-score of survival fraction approximating a normal distribution of all 3 siRNAs for each gene (Fig. 1B). In high-throughput approaches, off-targets represent a significant source of false positive hits. Thus, to avoid off-target effects, only genes which had a z-score<-2 with at least 2 different siRNAs were considered and are displayed on the heat map (Fig. 1C). Among these genes, 5 hits that fulfill these criteria were selected for further validation: *AKAP12 (*A-kinase anchor protein), *CSNK1α1* (casein kinase 1, alpha 1), *EPHA7* (EPH receptor A7), and *PCTK1* (PCTAIRE protein kinase 1) (Fig. 1C). AKAP12 is a scaffolding protein in signal transduction associated with protein kinases A and C and is implicated in several cellular processes: cell migration, maintenance of cytoskeletal architecture and cell proliferation [15]. Casein kinase 1 is a monomeric serine-threonine protein kinase involved in a number of cellular processes including DNA repair, cell division, nuclear localization and membrane transport. CSNK1 isoforms have key roles in the developmentally important Wnt and Hedgehog (Hh) signaling pathways [16]. EPH receptor A7 belongs to the ephrin receptor subfamily of the protein-tyrosine kinase family and is involved in mediating developmental events, particularly in the nervous system [17]. PCTAIRE protein is a member of CDK family with a role in cell cycle and proliferation, vesicle trafficking, neurite growth and spermatogenesis [18].

### Validation of siRNA screen candidates

**Figure 2:**
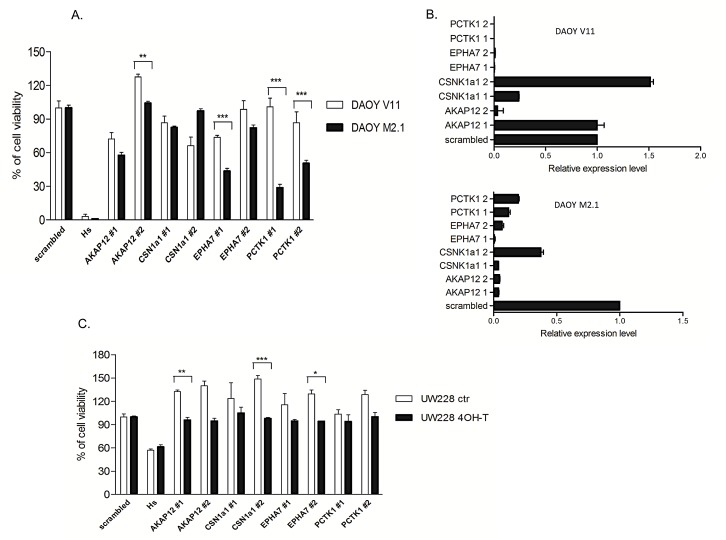
Validation of the screen A. siRNA knockdown repeated for the 4 hit candidates with two siRNA sequences in cells used in the screen. B. Validation of the siRNA transfection efficiency. Quantitative RT-PCR shows gene downregulation after 24 h of transfection with two different siRNA sequences. C. Knockdown of gene candidates in UW228 MycER cells. *p<0.05 when c-Myc basal level cells and c-Myc over-expressing cells are compared, error bars represent the SD of the mean of three independent experiments.

**Figure 3:**
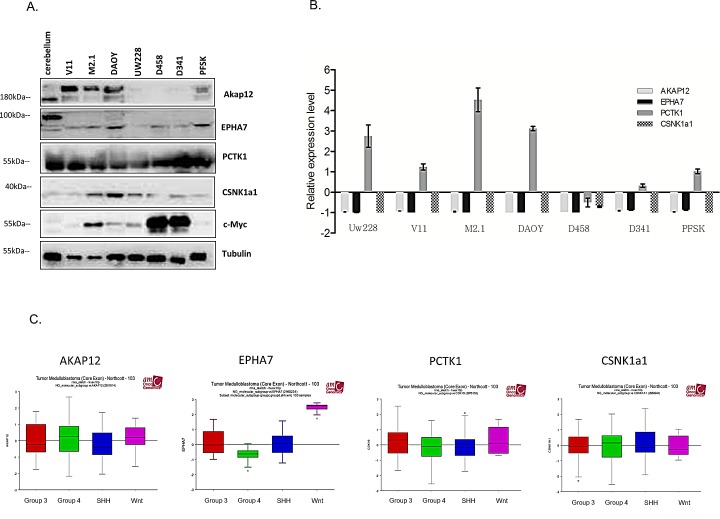
Expression panel of gene candidates in MB cell lines and patient samples A. Protein expression of gene candidates in healthy cerebellum (CB) and MB cell lines with and without c-Myc over-expression. B. Gene expression of gene candidates in CB and MB cell line panel. Expression level was normalized to CB (CB=0). C. r2 microarray analysis of gene expression candidates in molecular subgroups of MB.

To validate the results of the screen, all 5 candidate genes were silenced with two separate siRNA sequences (z-score <-2) in both DAOY clones and subsequently tested for viability according to the screen scheme. As expected, the cell viability of M2.1 cells was decreased after target gene silencing, when compared to V11 cells without c-Myc overexpression (Fig. 2A). To confirm knockdown efficiency two siRNA sequences were assayed for target down-regulation by quantitative RT-PCR. An analysis of gene expression levels indicated that both siRNAs efficiently silenced the corresponding target genes (Fig. 2B). To rule out that the c-Myc-depended cell viability decrease was limited only to DAOY clones, we tested the effect of the knockdown of the 5 gene candidates in another MB cell line: UW228 with inducible c-Myc-ER activation upon tamoxifen (4-OHT) treatment. Consistent with our previous observations, knockdown of the selected genes upon c-Myc induction led to sensitization of cells, although not to the same extent as in DAOY (Fig. 2C). Mentioned difference could be explained by various expression levels of PCTK1 in DAOY and UW228 cells, with lower levels in the last one. Subsequently we examined the expression profile of the candidate genes in MB cell lines with (DAOY M2.1, D458 and D341) and without (DAOY V11, DAOY, UW228, PFSK) c-Myc overexpression and healthy cerebellum (CB). Protein expression levels differed from each other in the panel of MB cells and CB (Fig. 3A). The gene expression analysis revealed that most of the candidates were down-regulated when compared to CB with PCTK1 being an exception as it was in the most cases up-regulated (Fig. 3B). Additionally, an analysis of gene expression in the microarray analysis and visualization platform r2 (R2: microarray analysis and visualization platform: http://r2.amc.nl) did not reveal any significant differences between molecular subgroups of MB, with EPHA7 as an exception (Fig. 3C).

To assess whether any of the 5 candidate genes were potential transcriptional targets of c-Myc, we first analyzed DNA microarray data from DAOY clones [19]. There were no significant changes in the expression of the 5 candidate genes between V11 and M2.1 cells (data not shown). We subsequently analyzed Chip data from MB cells [20] and found that only EPHA7 is a direct transcriptional target of c-Myc.

### Pharmacological inhibition of PCTK1 with a newly synthetized inhibitor

**Figure 4:**
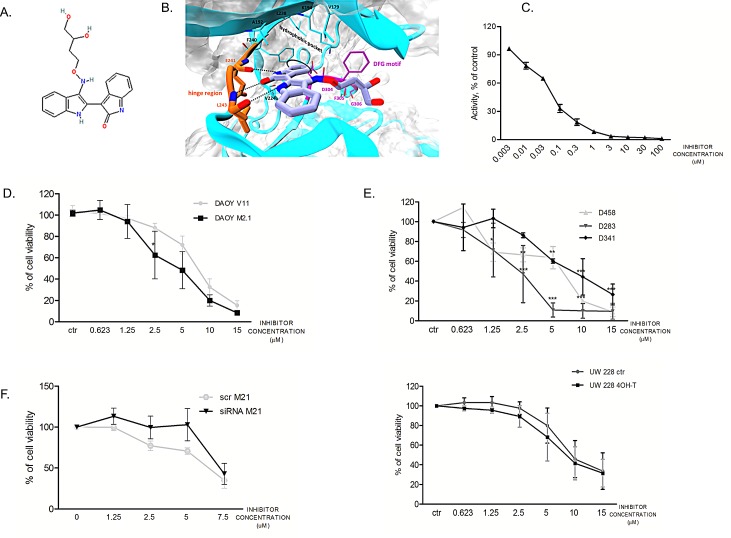
Synthesized PCTK1 inhibitor and its effect on MB cell lines A. Chemical structure of the PCTK1 inhibitor, indirubin E804. B. 3D cartoon of indirubin E804 binding into PCTK1 kinase domain visualized by Visual Molecular Dynamics (VMD) - molecular visualization program. The inhibitor's carbon atoms are in light blue while the hinge-region ones are in orange. The h-bonds are shown as black dotted lines. The DFG motif is in magenta while the rest of the protein is in cyan. C. A radiometric kinase assay (33PanQinase® Activity Assay) of the PCTAIRE1/CycY protein kinase activity. D. Inhibitor titration in DAOY V11 (grey circles) and M2.1 cells (black squares) E. Inhibitor titration in panel of MB cell lines (upper and lower panel). F. Inhibitor titration in DAOY M2.1 cells transfected with siRNA against PCTK1 (black triangles) or scrambled siRNA (grey circles).

Considering the highest degree of cell viability decrease upon target gene silencing, we chose to focus on PCTK1 in our further investigations. PCTK1 kinase is among the most poorly characterized CDK family members with no evidence of an implication in cancer. A PCTK1 inhibitor (indirubin E804) was synthesized based on a structure available in the Drugbank: http://www.drugbank.ca/drugs/DB07766 (Fig. 4A). By the means of Visual Molecular Dynamics (VMD) [21] the analysis of the crystallographic structure of PCTK1 kinase domain in complex with indirubin-based inhibitor (PDBcode: 3MTL) revealed that the inhibitor binds to the ATP site by interacting with the hinge region via H-bond interactions (Fig. 4B). In particular, the following hydrogen-bonds were identified: (i) the ligand NH group, belonging to the indole-2-one moiety, H-bonded to E241 backbone (3.08 Å); the carbonyl group of the same moiety interacted via H-bond with the NH-amide of L243 backbone (2.88 Å); (iii) the NH group, belonging to the indole moiety, also formed an H-bond with the carbonyl oxygen of L243 backbone (2.89 Å). Whereas the hydroxybutyloxime group is located in a solvent-exposed region, the phenyl ring of indole-2-one moiety turned out to be oriented in an opposite pocket establishing hydrophobic contacts with V224, F240, A192, V179 and F305. Other hydrophobic contacts within a maximum distance of 3.90 Å have been identified by using LigPlot [22] (Supplementary figure S2) particularly between the indole ring of the inhibitor and the side chains of residues L171 and L293. Notably, focusing the attention on the DFG motif, in contrast with the CDK2 structure bound to cyclin A2 and complexed with the ATP (D_in_ nad F_out_)(PDBcode: 1FIN), [23, 24] the D304 and F305 residues are respectively fixed in the *out* and *in* positions confirming the Indirubin-mediated block of PCTAIRE1 in the inactive state conformation (supplementary figure S2).

A radiometric protein kinase assay (33PanQinase® Activity Assay) was used for measuring the kinase activity of the PCTAIRE1/CycY protein kinase. The IC_50_ of the compound was determined to be 53nM ± 9.8nM *in vitro*, suggesting a relatively potent inhibitory activity of the inhibitor (Fig. 4C).

In order to determine the sensitivity of the two DAOY clones to the PCTK1 inhibitor dose-dependency studies were performed (Fig. 4D). c-Myc overexpressing cells (M2.1) were more sensitive to the PCTK1 inhibitor than those with basal c-Myc levels. A similar trend was shown in cells with endogenous c-Myc overexpression (D283, D458, D341) (Fig. 4E, upper panel) and in cells with inducible c-Myc – (UW228-Myc-ER treated with 4-OHT in comparison to un-induced cells) (Fig. 4E, lower panel), indicating a broad cytostatic effect of the inhibitor in c-Myc overexpressing MB cells. The statistical significance was evaluated: (1) between c-Myc basal level and c-Myc over-expressing cell lines; or (2) between different conditions and control.

In order to evaluate the specificity of the inhibitor toward PCTK1, we investigated whether silencing of PCTK1 could change the impact of the compound on the viability of DAOY M2.1 cells. A rescue in the viability of PCTK1-knock-down cells, without a comparable effect on the scrambled-transfected control cells was observed up to a 5 μM concentration of the compound, suggesting selectivity of the inhibitor towards PCTK1 (Fig. 4F).

### PCTK1 inhibition alters c-Myc expression and stability

**Figure 5:**
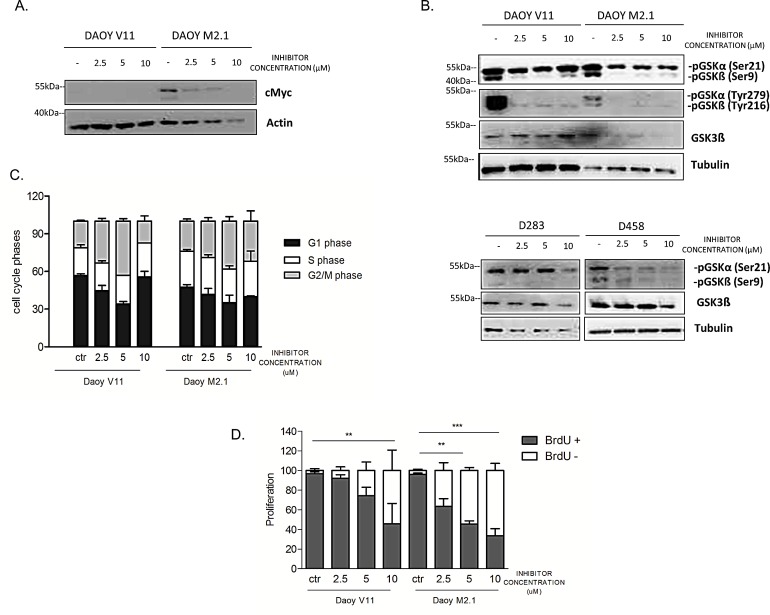
PCTK1 inhibition affects numerous cellular processes in c-Myc hallmarked MB cell lines A. c-Myc protein level upon PCTK1 pharmacological inhibition. B. Analysis of GSK3α/ß protein level upon treatment with PCTK1 inhibitor in MB cell lines. C. Cell cycle analysis was performed by PI (Propidium Iodide) DNA content measurement by FACS. G2/M arrest was observed upon inhibitor treatment independently of c-Myc status. D. BrdU incorporation measurement demonstrating cell proliferation decrease in dose dependent manner.

The observed effect of the inhibitor on c-Myc overexpressing cells led us to investigate whether PCTK1 inhibition has a direct influence on c-Myc protein levels. Indeed c-Myc protein expression decreased upon PTCK1 inhibitor treatment in a dose-dependent manner (Fig. 5A). In addition, PCTK1 inhibitor treatment impaired the phosphorylation of GSK-3α/β (Tyr 279/Tyr 216) in DAOY V11 and M2.1 cells (Fig. 5B). Interestingly, phosphorylation of GSK-3β at Ser9 was significantly deceased upon inhibitor treatment, which is an important observation relevant for c-Myc degradation. However, phosphorylation of GSK-3α at Ser21 remained unchanged. Nevertheless, there was no notable difference in the effect of the inhibitor on GSK-3 phosphorylation between the two cell lines (Fig. 5B, upper panel). Similarly, phosphorylation of GSK-3β at Ser9 was decreased upon PCTK1 inhibition in two MB cell lines with endogenous c-Myc overexpression (D283 and D458) (Fig. 5B, lower panel). Additionally, in one of the c-Myc overexpressing cells (D458) the phosphorylation levels of GSK-3 at Ser21 was decreased.

### Cell cycle and cell proliferation alteration upon PCTK1 inhibitor treatment

As PCTK1 is a member of CDKs and displays peak expression and maximal activity during the S-phase of the cell cycle [25], we investigated whether depletion of this kinase had an impact on cell cycle progression in MB. We analysed the stages of the cell cycle in untreated and treated populations of MB cells using PI uptake analyzed by FACS. We showed that PCTK1 inhibition resulted in G2/M arrest in both DAOY clones indicating its putative role in cell cycle in MB, independently of c-Myc (Fig.5C). However, the effect was abolished at the highest concentration of the inhibitor due to elevated cell death. Subsequent analysis of the effect of PCTK1 inhibition on newly synthesized DNA in replicating cells provided a possible elucidation of synthetic lethality identified in the RNAi screen. An analysis of BrdU incorporation during the S-phase of the cell cycle showed that depletion of PCTK1 led to decreased cell proliferation in a dose-dependent manner in the c-Myc-overexpressing MB cell line (Fig. 5D).

### PCTK1 inhibition affects the AKT/mTOR signaling pathway and induces cell death

**Figure 6:**
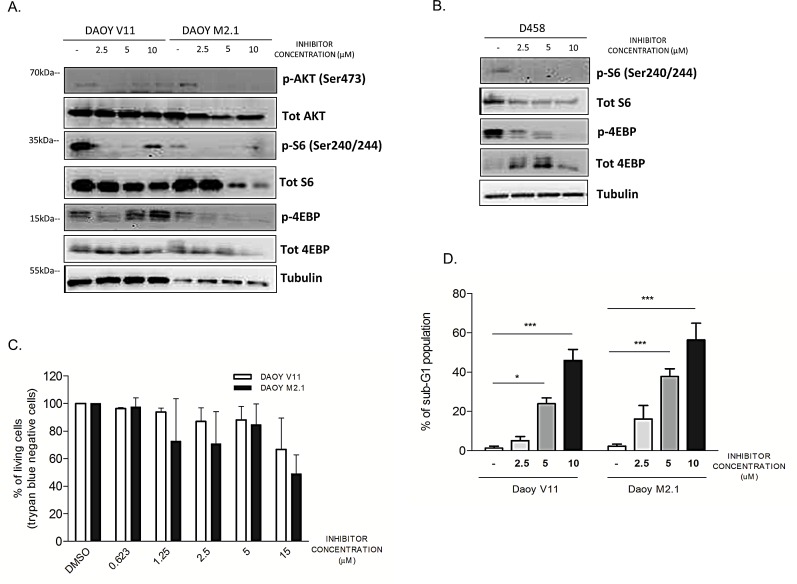
PCTK1 inhibition alters Akt/mTOR pathway in c-Myc hallmarked MB cell lines A. Analysis of signaling pathway regulators protein level upon treatment with PCTK1 inhibitor in DAOY V11 and M2.1 and B. in D458 cells C. PCTK1 inhibition induces cell death in dose dependent manner as shown by trypan blue staining and D. sub-G1 cell accumulation.

To gain further insight into the mechanism of action of the compound, we investigated whether inhibition of PCTK1 had an influence on survival signalling pathways upstream of c-Myc. Among the signaling components altered by PCTK1 inhibition in c-Myc-overexpressing cells (DAOY M2.1 and D458), we identified p-4E-BP and p-S6 (Fig. 6A and B). This indicates that the inhibitor affects the Akt/mTOR pathway as 4E-BP and S6 protein are targets of mTOR, which plays a key role in protein translation and cell growth [26]. Interestingly, the expression levels of these proteins increased in V11 cells when treated with high concentration of the PCTK1 inhibitor (10μM), suggesting the existence of a positive feedback loop (Fig. 6A). Phosphorylation of Akt (Ser473) was diminished in M2.1 cells, while higher concentrations of the PCTK1 inhibitor restored phosphorylation levels in DAOY V11 cells.

There is evidence that mTOR inhibition impairs the proliferation and survival of c-Myc-overexpressing cancer cells, but this inhibition does not directly involve an effect on expression of the c-Myc protein [27]. We could confirm this observation in MB cell lines by showing that c-Myc sensitizes MB cells to mTOR inhibition with PP242 [28]. However, c-Myc protein levels did not change markedly upon PP242 treatment (Supplementary figure S3).

To determine whether PCTK1 inhibition also induces cell death, we performed a Trypan Blue dye exclusion test upon PCTK1 inhibitor treatment. c-Myc over-expression sensitized MB cells for PCTK1 inhibition as more trypan blue positive cells (non-viable) appeared in DAOY M2.1 in comparison to V11, particularly at lower concentrations (Fig. 6C). Additionally, Fluorescence Activated Cell Sorting (FACS) analysis revealed a pronounced accumulation of M2.1 cells with hypodiploid DNA content (sub-G1) in a dose-dependent manner; whereas only a minor accumulation of sub-G1 population was observed in V11 cells (Fig. 6D).

### PCTK1 Inhibitor blocks tumour growth in a Chicken Chorio-Allantoic membrane (CAM) model of c-Myc overexpressing MB

**Figure 7:**
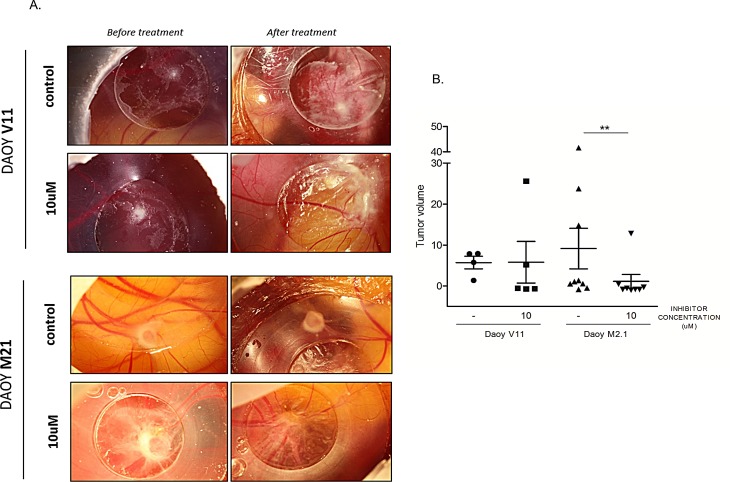
Chick Chorioallantoic Membrane (CAM) tumor growth assay as a model to study PCTK1 inhibition *in vivo* A. DAOY V11 or M2.1 – derived tumors formed on (CAM) were treated with dimethylsulphoxide (DMSO) (control) or 10 μM of inhibitor. B. Quantification of tumor volume before and after treatment is shown. Lines indicate the mean of each group.

The potential effect of pharmacological PCTK1 inhibition on tumor growth was examined *in vivo* using the Chorio-Allantoic Membrane (CAM) assay. Inoculation of DAOY V11 and M2.1 cells on the CAM resulted in the formation of solid tumors within three days (Fig. 7A). Notably, tumors formed by implantation of DAOY M2.1 grew more in comparison to DAOY V11, which is consistent with the commonly known phenomenon that tumors overexpressing c-Myc are characterized by a more aggressive phenotype. Treatment of tumors for 4 consecutive days with the PCTK1 inhibitor (10 μM) significantly impaired tumor growth in c-Myc overexpressing tumors, when compared to control (DMSO treatment) (V<0) (Fig. 7A,B). Importantly, the inhibitor was very well tolerated in chicken embryo without noticeable toxicity (data not shown).

## DISCUSSION

There exist so far few validated molecular targets for c-Myc-amplified cancers and medulloblastoma in particular. Intriguingly, the subgroup of MB characterized by activated SHH signalling is perhaps the one in which most targets have been described so far. The direct targeting of c-Myc in MB has been described by various approaches, including RNAi and pharmacological approaches [29, 30, 31]. In addition, it has been reported that interfering with signalling pathways controlled by c-Myc can impair MB cell proliferation [11, 32].

Loss-of-function screens have been used to identify synthetic lethal interaction partners of various oncogenes, including Myc [10]. The present study has identified several synthetic lethal interactions between c-Myc and protein kinases in MB. The kinases identified in the RNAi screen were apparently not direct targets of c-Myc (with the exception of EPHA7) and did generally not show any selectivity in terms of expression in the four molecular subgroups of MB.

PCTAIRE kinases belong to a highly conserved subgroup of cyclin-dependent kinases, which generally remain poorly characterized. Vertebrates possess three highly similar PCTK isoforms (PCTK1,2,3, also termed CDK16,17,18), which are most abundantly expressed in post-mitotic cells in brain and testis [24]. The highest expression levels of PCTK1 are found in the pyriform cortex, hippocampus and cerebellum [33, 34, 35]. The selective enrichment of PCTK1 expression in brain may reflect its role in MB. A recent report has demonstrated a link between PCTK1 and tumorigenesis. PCTAIRE1 was shown to play an essential role in the mitosis of tumor cells and the tumor suppressor p27 was discovered as a novel substrate of PCTK1. An evaluation of PCTK1 expression levels in primary tumors showed that its expression was significantly elevated in prostate and breast cancer [36].

PCTK1 belongs to group of proteins containing carboxy-terminal sequence binding PDZ (PSD95-Discs Large-Zonula Occludens) domains. PDZ domains containing proteins are involved in many different cellular processes and disturbance of PDZ networks is observed in many pathologies, including cancer [37, 38]. A two-hybrid screen in yeast described new potential interaction partners of PCTK1: SNX27, MAGI3 and PTPN3 [39]. Other proteins were also reported as substrates of PCTK1, including MBP (Myelin Basic Protein) and Histone H1 [40, 33]. Several studies presented the possible mode of activation and function of PCTAIRE-1 in cells. Firstly, PCTAIRE is phosphorylated by PKA (Protein Kinase A) at four sites in its N-terminal region, with S153 being an inactivating site. [41]. CDK5-mediated phosphorylation at S95 enhances PCTK1 activity [40]. PCTAIRE kinase 1 is activated by cyclin Y (CCNY), a highly conserved single cyclin [24, 25].

PCTK1 inhibition in MB resulted in decreased DNA synthesis and cell death. Initially, we determined whether apoptosis is the process responsible for death of MB cells. However, we found no evidence of apoptosis upon PCTK1 inhibition, which confirms previous reports that PCTK1 does not play a role in apoptosis [41].

The simplest model accounting for the selective effect of the PCTK1 inhibitor on the proliferation of MB cell lines over-expressing c-Myc is that this compound down-regulates c-Myc expression. Indeed, a previous report has documented that RNAi-mediated silencing of c-Myc impairs cell proliferation in MB cell lines over-expressing the oncogene [30]. The observation that PCTK1 inhibitor treatment impairs GSK-3β phosphorylation on Ser9 provides a possible link between PCTK1 and c-Myc at the molecular level. Indeed, PCTK1 phosphorylates GSK-3-derived peptides *in vitro*. Phosphorylation of GSK-3β at Ser9 decreases its activity, thus inhibition of this phosphorylation upon PCTK1 inhibitor treatment increases GSK-3β activity leading to degradation of c-Myc.

Several recent reports have documented that targeting PI3K/mTOR pathway can impair the proliferation and survival of MB, including mouse models of c-Myc-amplified MB [42, 43]. In neuroblastoma, another embryonal tumor, in which amplification of *MYC* family genes is correlated with poor prognosis, targeting PI3K/mTOR resulted in down-regulation of *MYCN* expression. Tuberous sclerosis complex (TSC) proteins may represent a putative link between PCTK1 and the mTOR pathway. Both TSC2 and TSC1 are regulated by phosphorylation [44]. TSC1 is phosphorylated at multiple sites by cyclin-dependent kinase 1 (CDK1)/cyclin B during the G_2_/M phase of the cell cycle [45].

Together, our findings indicate that PCTK1 is a new molecular target in c-Myc-overexpressing MB and suggest a therapeutic option to improve the treatment of high-risk MB patients.

## MATERIAL AND METHODS

### Cell culture and reagents

Medulloblastoma (MB) cells were purchased from the American Type Cell Culture Collection. DAOY, D283, D328, D458 and DAOY V11 and DAOY M2.1 cells were cultured in Improved MEM medium (Invitrogen, Carlsbad, CA, USA) supplemented with 1% L-glutamine (Invitrogen, Carlsbad, CA, USA), 1% Penicillin/Streptomycin (Invitrogen Carlsbad, CA, USA), 10% fetal calf serum (DAOY, D458, V11, M2.1) and 20% fetal calf serum (D238). Antibiotic G418 (Promega, Madison, WI, USA) was added in concentration 500μg/ml to DAOY clones (V11 and M2.1) for selection. UW228-MycER and PFSK cells were cultivated in Dulbecco's modified Eagle's medium (Sigma-Aldrich, Buchs, Switzerland) supplemented with 1% L-glutamine (Invitrogen, Carlsbad, CA, USA), 1% Penicillin/Streptomycin (Invitrogen, Carlsbad, CA, USA) and 10% fetal calf serum (Invitrogen, Carlsbad, CA, USA). 1 μmol/L 4-hydroxytamoxifen (4OH-T) (Sigma-Aldrich, Buchs, Switzerland) was added to culture medium of UW228-MycER cells to induce Myc expression.

### High throughput siRNA screen (HTS) design and validation

The medulloblastoma DAOY cell line with basal level of c-Myc was engineered to stably overexpress c-Myc, as described herewith [14]. DAOY clones V11 and M2.1 were plated in triplicates at 5000 cells per well in 96-well plates and transfected with 3 different siRNA targeting 719 protein and lipid kinases (Silencer Kinase siRNA Library Ambion, Applied Biosystems, Foster City, CA, USA) at a final concentration of 40 nM, using Lipofectamine 2000 according to the manufacturer's instructions (Invitrogen, Carlsbad, CA, USA). Additional controls were added to each plate of the library: negative control - siCONTROL non-targeting siRNA Pool (Dharmacon, Waltham, MA, USA) and positive controls: siRNA for PLK1 and AllStars Hs Cell Death Control siRNA (Qiagen, Venlo, Netherlands). At 48 h following transfection cell viability was assessed using MTS ((3-(4,5-dimethylthiazol-2-yl)-5-(3-carboxymethoxyphenyl)-2-(4-sulfophenyl)-2H-tetrazolium) assay (Promega, Madison, WI, USA). The evaluation of the HTS was utilized by median centered Z-scores of the surviving fractions calculation as described in Zhang et al [46]. Two distinct siRNA sequences targeting each candidate hit kinase were used to validate results from the screen (AKAP12: siRNA IDs 137856 and 137858; CSNK1a1: siRNA IDs 178 and 179; EPHA7: siRNA IDs 103321 and 527; PCTK1: siRNAs ID 1566 and 1472 (Ambion, Applied Biosystems, Foster City, CA, USA). Validation of RNAi gene silencing was evaluated 48 h after transfection by western blotting for protein expression and by semi-quantitative PCR for gene expression.

### Western blotting

Protein expression was analyzed by western blotting as described in Arcaro et al. [47]. The following antibodies were used: cMyc, p-AKT (Ser473), p-4E-BP1 (Thr37/46), p-S6 Ribosomal Protein (Ser240/244) (Cell Signaling Techology, Danvers, MA, USA), Ephrin receptor A7, casein kinase I α, PCTAIRE-1, GSK-3ß, p-GSK-3α/ß (Tyr 279/Tyr 216) (Santa Cruz Biotechnology, Santa Cruz, CA, USA), A kinase (PRKA) anchor protein 12 (Epitomics, Burlingame, CA, USA), ß-actin and α-tubulin (Sigma, Buchs, Switzerland). Quantification of protein expression was performed using TotalLab Quant (TotalLab Limited, UK).

### Quantitative RT-PCR

RNA was extracted using RNeasy Mini Kit (Qiagen, Venlo, Netherlands) following the manufacturer's instructions. The primers for 18S (5′-CCTCCAATGGATCCTCGTTA-3′ R, 5′-AAACGGCTACCACATCCAAG-3′ F), ß-actin (5′-AGCACTCTGTTGGCGTACAG-3′ R, 5′-GGACTTCGAGCAAGAGATGG-3′ F), AKAP12 (5′-CATGGCTCCTCCGCACTTCTC-3′ R, 5′-GTCTCCTTCATTCGCAGGCT-3′ F), EPHA7 (5′-AGGTCCGTTCCCTTTGATCT-3′ R, 5′-AGAGGCTCTTTGCTGCTGTC-3′ F), PCTK1 (5′-GTTTCCCAAAGCCAATCTCA-3′ R; 5′-GGAGAGTGACCAGGCTTCAG-3′ F) and CSNK1α1 (5′-TGTTGCCTTGTCCTGTTGTC-3′ R; 5′-GGATCTTCTGGGACCTAGCC-3′ F) were purchased from Microsynth (Balgach, Switzerland). 18S and ß-actin genes were used as housekeeping genes. cDNA was transcribed using the High Capacity cDNA Reverse Transcription Kit (Applied Biosystem, California, USA) under the following condition: 25°C for 10min, 37°C for 120min and 85 °C, 5sec. Quantitative RT-PCR with SYBR green detection was performed on a ViiA™ 7 Real-Time PCR System (Applied Biosystem, California, USA) using the following condition: 50°C for 2min, 95°C for 10min followed by 40 cycles of: 95 °C for 15sec and 60°C for 1min; and 95°C for 15sec, 60°C for 1min and 95 °C for 15sec. Average fold changes were calculated by differences in threshold cycles (C_t_) between samples.

### Trypan Blue dye exclusion test

After 24h treatment with inhibitor cells re-suspended in PBS were mixed with 0.1% trypan blue dye (Buchs, Switzerland, Sigma) in dilution 1:1. Cells were counted using a hemocytometer under a light microscope. Viable and nonviable cells were recorded separately, and the means of two independent cell counts were pooled for analysis. Percentages of living cells compared to the control were calculated.

### Protein kinase assay

A radiometric protein kinase assay (33PanQinase® Activity Assay) was performed by ProQinase Targeting Cancer to measure the kinase activity of the PCTAIRE1/CycY protein kinase. The reaction was executed in 96-well plate by mixing following reagents: 10ul non-radioactive ATP solution, 25 μl of assay buffer/[γ-^33^P]-ATP mixture, 5 μl of test sample in 10% DMSO and 10 μl of enzyme/peptide substrate GSK3(14-27) mixture. Protein kinase was expressed in Sf9 insect cells or in E.coli as recombinant GST-fusion proteins or His-tagged proteins was produced from human cDNA. The reaction was incubated at 30° C and was stopped after 60 minutes with 50 μl of 2 % (v/v) H3PO4, plates were aspirated and washed twice with 200 μl 0.9 % (w/v) NaCl. Incorporation of 33Pi (counting of “cpm”) was determined with a microplate scintillation counter (Microbeta, Wallac). The residual activity (in %) was calculated by using the following formula: $\mathrm{Res}.\;\mathrm{Activity}(\%)=100\frac{\mathrm{cmp of compound}-\mathrm{low control}}{\mathrm{high control}-\mathrm{low control}}$. IC50 values were calculated in Prism 5.04 using mathematical model “Sigmoidal response (variable slope)“.

### Flow cytometry

Fluorescence activated cell sorting (FACS) was employed to assess cell cycle, cell death and proliferation. Briefly, to assess cell cycle DAOY V11 and M2.1 cells were seeded at 2×10^5^ cells per well in 6-well plates and treated with PCTK1 inhibitor and vehicle (DMSO). After 24h treatment cells were collected and fixed in ice-cold 100% ethanol overnight. Cell pellets were re-collected and re-suspended in 40μg RNase A RNase A/50μg Propidiumiodide (Buchs, Switzerland, Sigma) cocktail in dark at 4°C for 30min. Proliferation of DAOY cells were assessed by bromodeoxyuridine (BrdU) incorporation using FITC BrdU Flow Kit (BD Pharmingen, Allschwil, Switzerland) according to the manufacturer's instruction.

Flow cytometric determination of DNA content, BrdU incorporation and sub-G1 population was conducted by LSRII Flow Cytometer (BD Pharmingen, Allschwil, Switzerland) and analyzed by FlowJo software (Tree Stars, Ashland, OR, USA).

### Chick chorio-allantoic membrane (CAM) assay

Fertilized chicken eggs (*gallus gallus*) from a local hatchery were incubated in a humidified incubator at 37°C. On day 3 (D3), a window was opened in the egg shell to prevent attachment of the chorio-allantoic membrane to the shell, and covered with tape to continue incubation. On D7, a silicon ring was placed on the CAM and 5×10^6^ DAOY V11 and M2.1 cells suspend in 20 μl PBS were applied after gentle laceration of the membrane. On D10, photos were taken under a Leica M205 FA microscope (10 x magnification) supplied with a Canon EOS 5D Mark II camera (Canon EOS Utility software) followed by tumors treatment for the four constitutive days with 20 μl of 15uM PCTK1 inhibitor or vehicle (DMSO). On D14, post-treatment photos were acquired and tumor volume was estimated by $V=\frac{4}{3}\pi r^{3}$, where $\text{r}=\frac{1}{2}\sqrt{d*d}$ [48].

### Chemical synthesis of PCTK1 inhibitor

Synthesis reactions and HPLC chromatograms are displayed in supplementary figure S1. Chemical synthesis of inhibitor was prepared according to a previously reported procedure [49]. Briefly, to a solution of indoxyl acetate 2 (5.00 g, 28.5 mmol) in anhydrous methanol (70 mL) under nitrogen was added isatin 1 (4.20 g, 28.8 mmol) and sodium carbonate (6.50 g, 61.3 mmol). After 30 min of stirring and standing for 24 h, slurry was filtered and washed with cold methanol and cold water. Obtained indirubin 3 (2.50 g, 9.5 mmol) and hydroxylamine hydrochloride (1.7g, 24.7 mmol) were refluxed together in pyridine (60 mL) for 1.5 h. Subsequently, mixture was poured into a 1M solution of hydrochloric acid (100 mL). The red/orange precipitate was filtered off, re-dissolved in 1M sodium hydroxide (70 mL) and re-precipitated with 1M hydrochloric acid (150 mL). The crude product was filtered, dried and recrystallized from ethanol/water (7:2) to give the product as bright red/orange crystals with spectral data in agreement with previously reported literature. In the end, Indirubin-3’-oximine 4 (55.4 mg, 0.20 mmol) and 4-bromobutane-1,2-diol 5 (40.1 mg, 0.24 mmol) were dissolved in anhydrous dimethylformamide (1.5 mL). To this was added triethylamine (33 μL, 0.24 mmol). The reaction was stirred under nitrogen for 4 h before the solvent was removed in vacuo to produce a crude brown oil which was purified via column chromatography to give the product as a red solid (36 mg, 41 % yield) with spectral data in agreement with previous reports [50].

### Structural Analysis

The 3D structural analysis was carried out by using the visualization program VMD [21] in order to examine the crystallographic complex of PCTK1/inhibitor within the ATP-binding site of kinase domain. In addition, the interactions of this complex have been investigated by the means of LigPlot [22] which provided a 2D schematic representation of H-bonds, hydrophobic and Van der Waals contacts.

### Statistical Analysis

All statistics were performed in GraphPad Prism software (La Jolla, CA, USA). The statistical significance between two groups was assessed with two-way ANOVA with Bonferroni's multiple comparison test and p < 0.05 was consider significant.

## SUPPLEMENTARY MATERIAL FIGURES


