# Supplementary material for: RNA interference screening identifies a novel role for PCTK1/CDK16 in medulloblastoma with c-Myc amplification
Source: Oncotarget. 2014 Nov 6;6(1):116–29. doi: 10.18632/oncotarget.2699 (PMC4381582; doi:10.18632/oncotarget.2699)
Supplement: Supplementary file 1 [file oncotarget-06-116-s001.pdf]

RNA interference screening identifies a novel role for PCTK1/CDK16 in medulloblastoma with c-Myc amplification

Supplementary Material

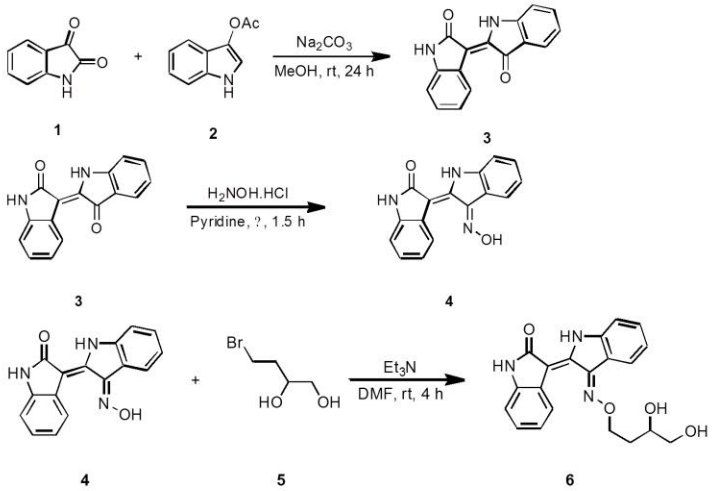

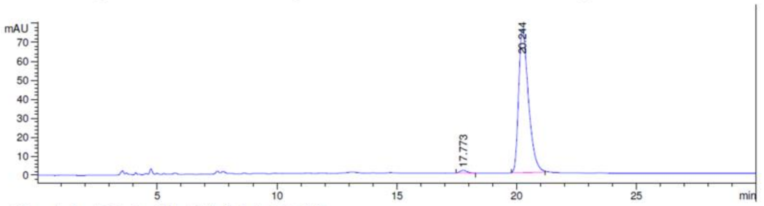

Signal 1: DAD1 A, Sig=254,8 Ref=360,100

Peak #	RetTime [min]	Type	Width [min]	Area [mAU*s]	Height [mAU]	Area %
1	17.773	BB	0.3147	28.24335	1.40399	1.2949
2	20.244	BB	0.4341	2152.94556	75.62990	98.7051
Totals :				2181.18891	77.03389	

Supplementary figure S1: Chemical synthesis of PCTK1 inhibitor - three-step chemical reaction aiming at synthetizing PCTK1 inhibitor: A. Synthesis of the inhibitor consists of three-step chemical reaction. Firstly, in presence of methanol and nitrogen indirubin (3) was produced. In the second step induribin was converted into indirubin-3´-oxime (4) by refluxion with hydroxylamine hydrochloride in pyridine. Subsequently this product was dissolved together with 4-bromobutane-1,2-diol in anhydrous dimethylformamide and triethylamine was added. After 4h reaction under nitrogen, column chromatography was performed and resulted in indirubin-3´-(2,3-dihydroxypropyl)oximether production (6). B. High-performance liquid chromatography (HPLC) chromatogram showing peak of synthetized inhibitor with its retention time around 20min.

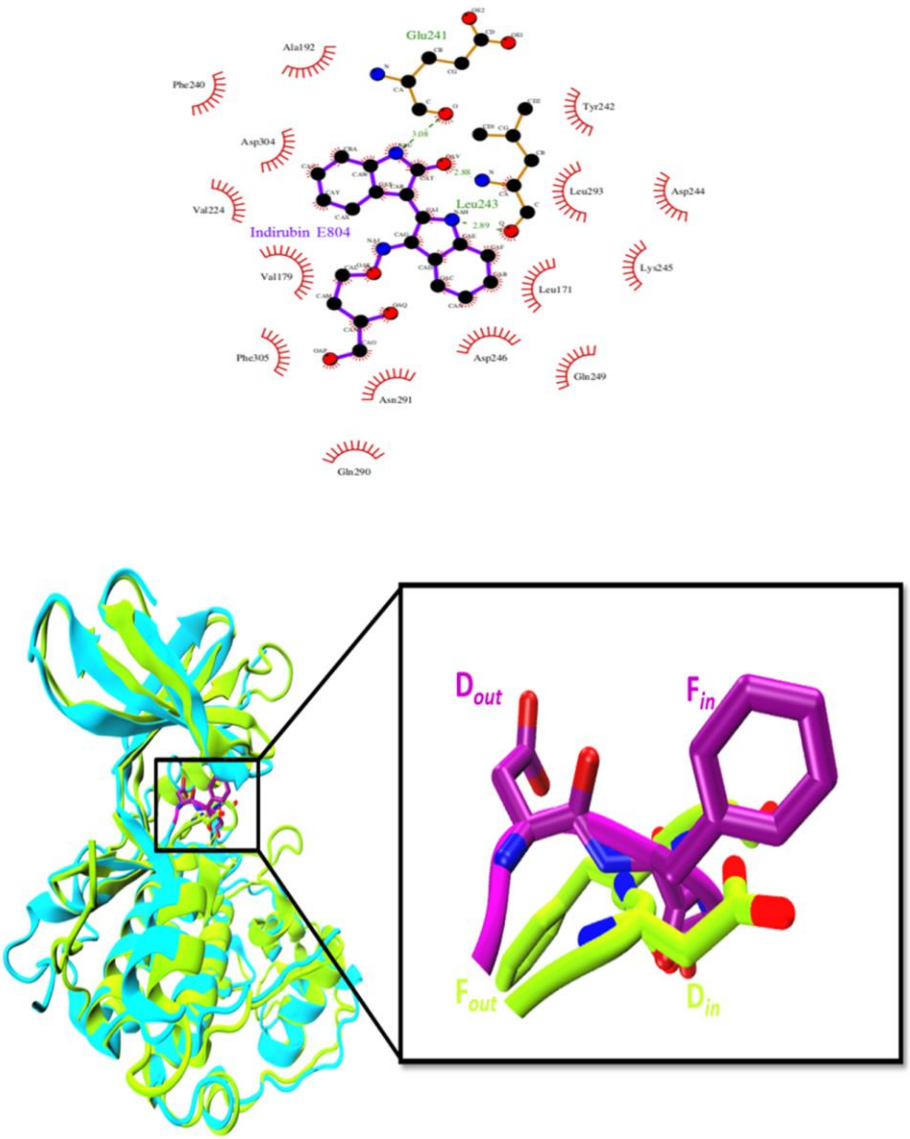

Supplementary figure S2: LigPlot analysis of inhibitor and the binding site residues of PCTAIRE1 kinase domain. A. Schematic representation of contacts between the inhibitor and the binding site residues of PCTAIRE1 kinase domain. The H-bond interactions between the inhibitor (iceblue) and the hinge region residues, E214 and L243 (orange), are highlighted in dashed green lines. The PCTAIRE1 binding site residues establishing hydrophobic and Van der Waals contacts with the Indirubin E804 within a maximum distance of 3.90 Å are also displayed. B. Overlap between the PCTAIRE1 kinase domain (light cyan) (PDBcode: 3MTL) and in the CDK2 structure (light yellow) (PDBcode: 1FKIN) and comparison of DFG motif locations in the two proteins (purple and light yellow respectively).

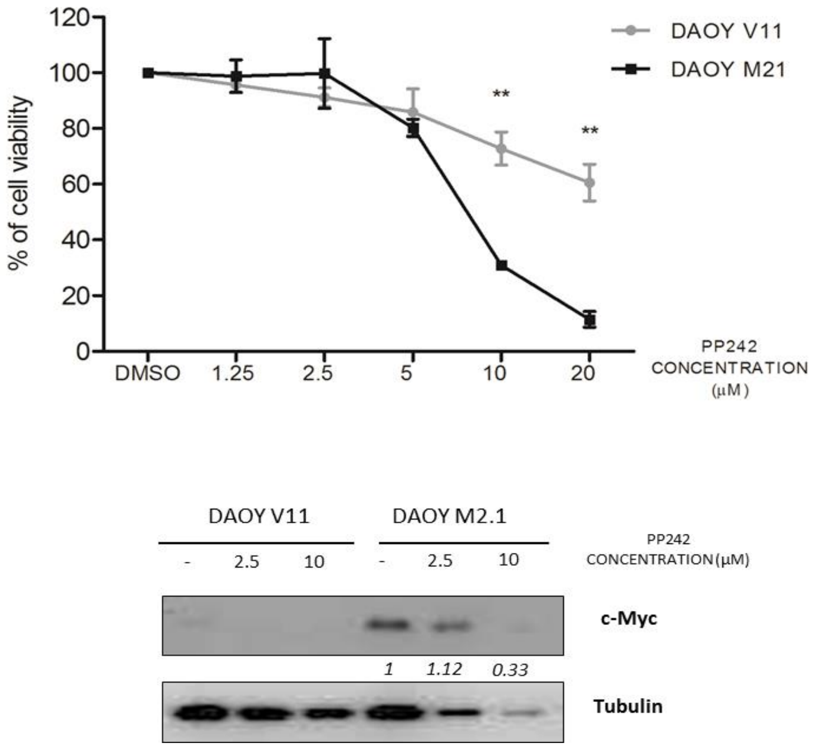

Supplementary Figure S3: c-Myc over-expression sensitize MB cells to mTOR inhibition. A. Cell viability assessment of DAOY V11 and M2.1 cell lines upon mTOR inhibition with PP242. B. Western blot analysis of c-Myc protein levels in M2.1 cells upon mTOR inhibition with PP242.
